# A Review of X-ray Photoelectron Spectroscopy Technique to Analyze the Stability and Degradation Mechanism of Solid Oxide Fuel Cell Cathode Materials

**DOI:** 10.3390/ma15072540

**Published:** 2022-03-30

**Authors:** Mustafa Anwar, Muhammed Ali Shaikh Abdul, Uneeb Masood Khan, Muhammad Hassan, Asif Hussain Khoja, Andanastuti Muchtar

**Affiliations:** 1 U.S.-Pakistan Center for Advanced Studies in Energy, National University of Sciences and Technology, H-12, Islamabad 44000, Pakistan; mustafa@uspcase.nust.edu.pk (M.A.); uneeb003@gmail.com (U.M.K.); hassan@uspcase.nust.edu.pk (M.H.); asif@uspcase.nust.edu.pk (A.H.K.); muchtar@ukm.edu.my (A.M.); 2 Fuel Cell Institute, Universiti Kebangsaan Malaysia, Bangi 43600, Malaysia; 3 Department of Materials and Manufacturing Engineering, Faculty of Engineering and Built Environment, Universiti Kebangsaan Malaysia, Bangi 43600, Malaysia

**Keywords:** solid oxide fuel cell, cathode, degradation, X-ray photoelectron spectroscopy, characterization

## Abstract

Nondestructive characterization of solid oxide fuel cell (SOFC) materials has drawn attention owing to the advances in instrumentation that enable in situ characterization during high-temperature cell operation. X-ray photoelectron spectroscopy (XPS) is widely used to investigate the surface of SOFC cathode materials because of its excellent chemical specificity and surface sensitivity. The XPS can be used to analyze the elemental composition and oxidation state of cathode layers from the surface to a depth of approximately 5–10 nm. Any change in the chemical state of the SOFC cathode at the surface affects the migration of oxygen ions to the cathode/electrolyte interface via the cathode layer and causes performance degradation. The objective of this article is to provide a comprehensive review of the adoption of XPS for the characterization of SOFC cathode materials to understand its degradation mechanism in absolute terms. The use of XPS to confirm the chemical stability at the interface and the enrichment of cations on the surface is reviewed. Finally, the strategies adopted to improve the structural stability and electrochemical performance of the LSCF cathode are also discussed.

## 1. Introduction

**Figure 1:**
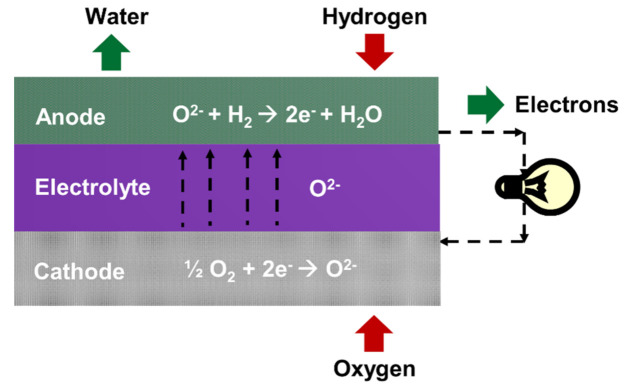
Schematic of solid oxide fuel cell.

Solid oxide fuel cells (SOFCs) are attractive and promising energy conversion devices for electricity production because of their high efficiency, fuel flexibility, and absence of precious noble metal catalyst [1,2,3]. A basic SOFC consists of a dense electrolyte, which is in contact with two porous and electro-catalytically active electrodes on either side (Figure 1) [4,5]. SOFCs operate at high temperatures (400–800 °C) and have numerous applications including residential, industrial, and transportation [5,6]. Currently, efforts are being made to reduce the operating temperature of SOFCs from 600 °C to 350 °C. Low temperature systems can save manufacturing cost by reducing the costs of insulation, materials, startup, and degradation. However, reduction of the operating temperature below 600 °C results in high electrolyte ohmic resistance and electrode polarization resistance, resulting in power loss [7,8,9]. These phenomena can be attributed to the physicochemical processes of the SOFC system, such as electrochemical reaction, heat conduction, transport of ions, electrons, and fuel, and convection [10]. Therefore, the reaction kinetics of individual components in SOFC contributes to the overall cell performance. The total cell electrical resistance of a single cell should be minimized to less than 0.25 Ω cm^2^ within the operating temperature range [11].

Identifying and understanding the influencing factors of these polarization losses is important to improve the overall SOFC cathode performance. These polarization losses can be minimized using the following methods: (1) identifying new promising cathode materials, (2) increasing the surface area of the cathode to enlarge the triple-phase boundary (TPB) connectivity, (3) increasing the reactant flow rate or pressure, and (4) adopting a tortuosity or a porous structure for the cathode to facilitate gas diffusion [12].

### 1.1. Different Classes of Perovskite-Based Cathode Materials for SOFC

**Figure 2:**
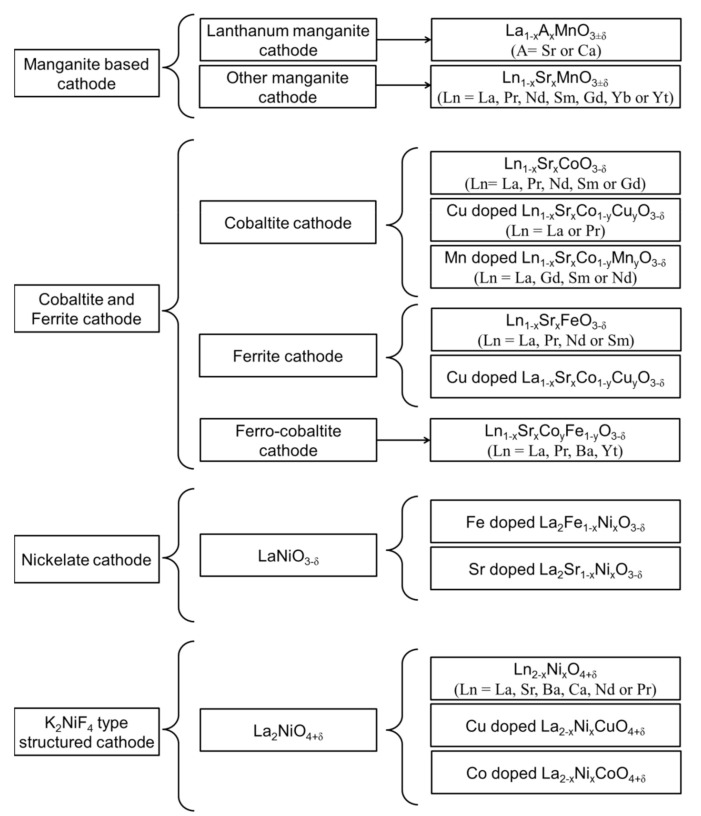
Comprehensive list of various perovskite-based cathode materials used in SOFCs.

Considering the high operating temperature and activation energy associated with the ORR on the cathode side of an SOFC, the choice of cathode is limited to platinum, gold, and silver [13]. However, noble materials are unsuitable for practical applications because of their high cost, poor compatibility with the electrolyte, and generation of a volatile oxide (PO_x_) during high-temperature oxidation [14]. Therefore, perovskite-structured materials have been widely used as cathode materials in SOFCs. A list of various cathode materials used in SOFCs is presented in Figure 2. The most promising cathode for high-temperature SOFC is strontium-doped lanthanum manganite (LSM) [15]. However, the LSM cathode shows high polarization resistance at a reduced temperature because of its high activation energy for ORR, making it unsuitable for intermediate-temperature (IT) SOFCs below 800 °C [16]. Thus, alternative cathode materials for SOFCs operating at reduced temperatures have recently attracted considerable research attention.

Lanthanum strontium cobalt ferrite or La_1−x_Sr_x_Co_1.y_Fe_y_O_3−δ_ (LSCF) perovskite has received a lot of attention due to its excellent chemical stability, high electrical conductivity, and high catalytic activity toward ORR [17]. LSCF cathodes have remarkably high oxygen diffusivities and oxygen surface exchange coefficients of 7.32 × 10^−6^ cm^2^/s and 1.5 × 10^−3^ cm/s, respectively, at 800 °C [18]. La_0.6_Sr_0.4_Co_0.2_Fe_0.8_O_3−δ_ (LSCF6428) exhibits an excellent electronic conductivity of 330 S/cm and ionic conductivity of 0.008 S/cm at 600 °C [19]. LSCF is also an excellent electrocatalyst for ORR in various applications, including electrodes for solid-state electrochemical devices, oxygen sensors, and dense membranes for oxygen separation from air in coal gasification and oxy-fuel power plants [20]. Thus, LSCF can be used as a cathode for IT-SOFC applications.

### 1.2. Surface Composition-Performance Relationship of SOFC Cathodes

Compositional changes on the surface of the cathode plays an important role in accelerating the ORR rate at a reduced operating temperature [21]. Considering that the ORR rate is related to the cathode surface composition. It typically determines the cell efficiency at low temperatures [22], because at the cathode, the O^2−^ ions must be transported to the cathode/electrolyte interface through the sufficiently porous structure (≈30%) to complete the first half of the electrochemical reaction [23]. This transport of O^2−^ ions through the porous cathode must be continuous. However, any physical resistance to the transport of O^2−^ ions through the cathode results in a voltage loss.

The change in the surface composition and its detrimental effect on the performance and stability of the SOFC cathode can be elucidated by in situ characterization during high-temperature cell operation. The composition and the structural, morphological, mechanical, optical, and electrical properties of an SOFC cathode can be analyzed using various techniques. Each technique can analyze the intrinsic and extrinsic properties of SOFC cathode materials. Mechanical analyses of the SOFC cathode are generally limited and not critical because of the porous structure of the cathode materials utilized, which are not used as freestanding support. Many new advanced techniques have been adopted, and researchers have extensively studied the microstructural distortion and gradual degradation of the electrochemical performance of energy materials [24].

### 1.3. Advanced Characterization of SOFC Cathdoe by Using Electron Spectroscopic Techniques

The use of an important analytical method on critically significant SOFC technology is certainly timely. Appropriate in situ measurements of elemental composition and chemical state of the SOFC cathode materials will be highly valuable towards improving its life expectancy and SOFC technology performance and stability. Therefore, to understand the complexity of the SOFC cathode degradation, researchers have developed new in situ and non-destructive experimental techniques to further explore the stability and degradation mechanism of SOFC cathode during fuel cell operation [25]. X-ray photoelectron spectroscopy (XPS) is one of the best tools for understanding compositional changes on the surface and at the interface of the SOFC cathode [26]. This technique can reveal the submicron-sized elements formed on the surface and at the cathode/electrolyte interface of the SOFC cathode [27]. Therefore, this paper reviews the use of XPS for studying the compositional changes on the surface and at the cathode/electrolyte interface of LSCF cathode materials because LSCF is one of the most commonly used cathode materials [28].

However, the low surface electrocatalytic activity for the ORR at reduced operating temperature and microstructural instability of the LSCF cathode are among the major concerns during SOFC operations. The segregation of Sr cation and subsequent reaction with CO_2_ and YSZ to form insulating-phase SrCO_3_ at the surface and SrZrO_3_ at the LSCF/YSZ interface can considerably affect the electrochemical performance of LSCF cathode materials [29,30]. Therefore, the microstructural changes and structural stability of the LSCF cathode should be determined. LSCF cathode degradation and XPS technique has been increasingly studied in the indexed literature [31,32,33,34]. This review paper aims to address how XPS is applied to SOFC cathode for understanding its degradation mechanism in absolute terms. The working principle, application, and limitations of XPS are also presented. To understand the fundamental behavior of the LSCF cathode under real fuel cell conditions, we determined its ORR mechanism and the microstructural degradation of the LSCF cathode. Finally, the strategies adopted to improve the structural stability and electrochemical performance of the LSCF cathode are discussed.

## 2. X-ray Photoelectron Spectroscopy (XPS)

### XPS Principle and Experimental Details

**Figure 3:**
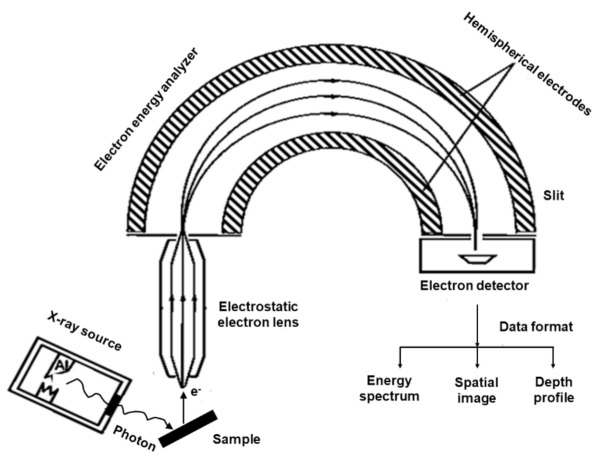
X-ray photoelectron spectroscopy experimental setup.

XPS is a well-established and widely used surface-sensitive spectroscopic technique for studying the elemental composition of the surface of SOFC cathode layers within the first few nanometers [2]. This photoelectron spectroscopic technique is used to quantitatively analyze the surface electronic structure of crystalline solid through the photoelectron spectra. The basic principle of the photoelectron effect was explained by Albert Einstein in 1905. Kai Siegbahn constructed an XPS instrument that can analyze photoelectron emissions and allow the speciation analysis of the sample surface. In XPS, the sample is irradiated by a soft X-ray (typically 1–3 keV) source (AlKα or MgKα) with an energy of *hν* to ionize electrons from the surface, as shown in Figure 3. The photoelectron penetrates the samples between 1 nm and 10 nm, where the photon is absorbed by an atom in the solid, leading to ionization and emission of the core electron in different directions. The atom then releases energy by the emission of an Auger electron, in which the L electron falls to fill the core-level vacancy. The KLL Auger electron is then emitted to conserve energy released during initial emission. The photoelectric effect generates free electrons with certain kinetic energy ($E_{kin}$) and is measured by a detector.

The number of photoelectrons emitted as a function of their kinetic energy ($E_{kin}$) can be measured using an electron energy analyzer, and the corresponding photoelectron spectrum can be recorded. The XPS instrument measures the kinetic energy of all collected electrons. The photoelectron spectrum includes the photoelectron and Auger electron lines. Each element produces a characteristic set of peaks in the photoelectron spectrum at particular binding energy (*E_B_*) values that directly identify the element on the surface of the sample analyzed. The XPS records the core-level values of the elements in electronvolt (eV). The XPS spectral lines are identified by the shell (e.g., 1s, 2s, and 2p), from which the electrons are emitted.

Typical XPS spectra represent intensity versus *E_B_*, where the intensity area reveals the concentration and binding energy reveals the speciation. The spectrum reveals the electron energy distribution in the material. The position and height (intensity) of each peak in the photoelectron spectrum provide the desired information on the chemical state, elemental composition, empirical formula determination, electronic state, and oxidation state of the sample surface. These data are used to determine the binding energy of the ejected electron to obtain information about the electronic structure using the equation:

(1) $$E_{B}=hv-E_{kin}-\Phi$$

where *h* is the Planck constant (6.63 × 10^−34^ J s), *ν* is the frequency (Hz) of radiation, *E_B_* is the electron binding energy, *E_kin_* is the kinetic energy of freed electrons, and Φ is the work function. Binding energy is an important parameter in the XPS analysis to obtain information about the electronic structure. The work function of the material is the difference between the Fermi level ($E_{F}$) and the vacuum level ($E_{vac}$). For an electron to be ejected or emitted from the solid, the energy of the photoelectron must be sufficient to overcome its attraction to the material or Φ of the material. Table 1 represents the binding energy levels from the ejected electron and their corresponding orbital from which the electrons are ejected for selected elements.

## 3. Characterization of LSCF Cathodes by Using XPS

### 3.1. XPS for Surface Segregation or Enrichment of Sr on the Surface

**Figure 4:**
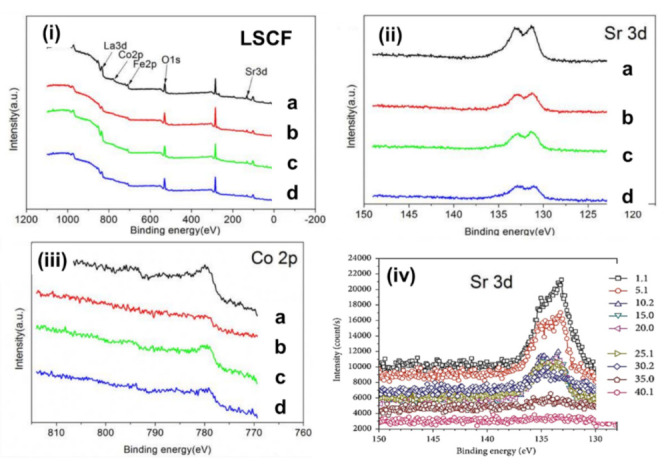
(**i**) XPS spectra of LSCF, showing La 3d, Sr 3d, Co 2p, and Fe 2p peaks (**ii**) magnified image of Sr 3d peak (**iii**) magnified image Co 2p peak for (a) as-prepared sample, (b) under open circuit at 750 °C in the air for 120 h, (c) polarized sample under 100 mA cm^−2^ at 750 °C for 120 h, and (d) polarized sample under 200 mA cm^−2^ at 750 °C for 120 h. (iv) Sr 3d peaks obtained from XPS depth profile measurements at different depths in micrometers (((**i**–**iii**) reprinted with permission from Reference [35], copyright Elsevier, 2018) and (**iv**) reprinted with permission from Reference [31], copyright Hindawi, 2018).

XPS provides details on the chemical composition near the surface region and the oxidation state of each element from the binding energy of particular core levels. Therefore, a highly surface-sensitive technique such as in situ synchrotron-based XPS can be used to understand how Sr segregation accelerates the degradation mechanism of the LSCF cathode, and this method often limits the surface catalytic activity for the ORR [2]. Figure 4 shows the typical XPS spectra of the LSCF cathode in the region with La 3d, Sr 3d, Co 2p, Fe 2p, and O 1s peaks. Liu et al. investigated the performance degradation mechanism of LSCF and LSCF/GDC composite cathodes under different cathodic current polarizations by using XPS (Thermo VG Scientific Multilab 2000, East Grinstead, UK) [35]. The XPS spectra indicate that the La/Sr ratio increased to 1.36 from 0.61 (under open-circuit voltage) after being polarized at the cathodic current of 100 mA cm^−2^ at 750 °C for 120 h. Therefore, the surface segregation or enrichment of Sr is favorable under the cathodic current polarization of 100 mA cm^−2^, but the concentration decreased to 0.76 after the current polarization of 200 mA cm^−2^ for 120 h. This decrease in the concentration of Sr on the surface implies the incorporation of Sr into the LSCF lattice under high cathodic polarization treatment. However, the La/Sr ratio after current polarization treatment remains smaller than the stoichiometric ratio of 1.5 for LSCF.

Table 2 summarizes the binding energy levels for LSCF cathode under different cathodic polarization treatments. The values of binding energies agree well with the values of La, Sr, Fe, and Co in LSCF (Table 1). No preferential change was observed in the binding energy for La 3d and Fe 2p peaks [35]. Hence, the valence state does not change under cathodic polarization treatment. However, the binding energy of Sr increased to 132.45 eV after current polarization at 200 mA cm^−2^ for 120 h, indicating the existence of Sr on the surface [Figure 4 (Sr 3d)] [35].

**Figure 5:**
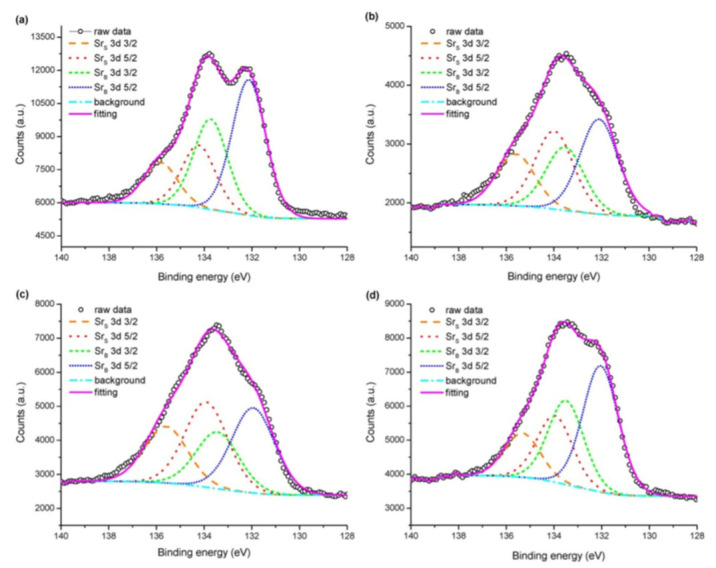
Peak fitting results of Sr spectra for (**a**) raw LSCF powder, (**b**) freshly prepared LSCF electrode, (**c**) LSCF electrode after 24 h annealing, and (**d**) nitric acid-treated LSCF electrode after annealing [36] (© The Electrochemical Society. Reproduced by permission of IOP Publishing. All rights reserved).

Pan et al. [36] studied the effect of Sr surface segregation of LSCF electrode on its electrochemical performance by using XPS. Peak fitting on XPS spectra of Sr was conducted to confirm the surface segregation of Sr based second phase other than that of LSCF perovskite and compared the XPS spectra of raw LSCF powder, freshly pre-pared LSCF electrode, LSCF electrode after annealing for 24 h, and nitric acid-treated LSCF electrode after annealing (Figure 5). The 3d spectrum of Sr shows the coupling of 3d_3/2_ and 3d_5/2_ spin orbits, resulting in a doublet state. Peak fitting of Sr 3d spectrum resulted in the extraction of two pairs of Sr 3d_3/2_ and Sr 3d_5/2_ spin orbits. The pair with lower binding energy was denoted by Sr_B_, which is located in bulk bound Sr, while Sr denotes the pair with higher binding energy present on the surface of the LSCF electrode.

### 3.2. XPS for Element Migration of Sr and Co

The XPS analysis shows that the atomic concentration of Co cation on the surface of LSCF increased from 6.91 (under OCV) to 9.93 after current polarization at 200 mA cm^−2^ for 120 h (Figure 4 [Co 2p])). This result indicates that cathodic current polarization promotes the migration of Co to the LSCF surface. This finding is consistent with the considerable decrease in the binding energy for the Co 2p peak for LSCF after cathode polarization resistance (Table 2). This phenomenon indicates the change in the oxidation state of Co from Co^2+^ to Co^3+^. Moreover, the performance degradation rate of the LSCF is higher in the high current density of 500 mA cm^−2^ at 750 °C than that in the lower current density. This result could be attributed to the accelerated migration of Sr and Co cations to the LSCF surface under high cathodic current polarization conditions, leading to the formation of the insulating SrCoO_x_ phase [37].

**Figure 6:**
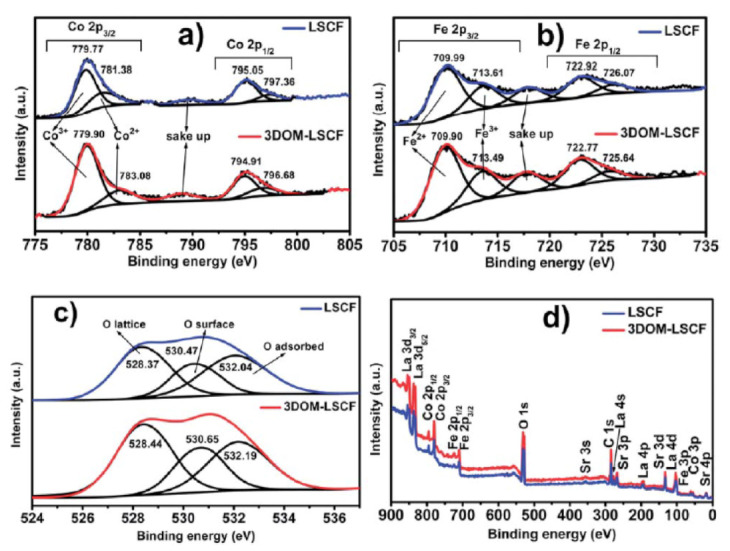
The XPS spectra of (**a**) Co 2p, (**b**) Fe 2p (**c**) and O 1s and (**d**) XPS survey spectra of the double perovskites LSCF and 3DOM-LSCF (Reproduced from Ref. [38] with permission from the Royal Society of Chemistry 2018).

Ha et al. [38] performed XPS of LSCF and 3DOM-LSCF (three-dimensionally ordered microporous LSCF) and found that both Co and Fe show two coexisting oxidation states (Figure 6). The B-site in the perovskite is occupied by Fe or Co in either 2+ or 3+ oxidation states. Furthermore, peak fitting for Fe 2p orbital was conducted within Fe^2+^ and Fe^3+^ component constraints and their respective satellites. The higher binding energy of iron denotes Fe^3+^ (Figure 6b). In addition, Co^2+^, Co^3+^, and their respective satellites were used to fit the Co 2p region. However, in this case, the higher binding energy is attributed to Co^2+^. The oxygen XPS spectra are shown in Figure 6c, which depict the lattice, surface, and adsorbed oxygen with increasing binding energy, respectively.

**Figure 7:**
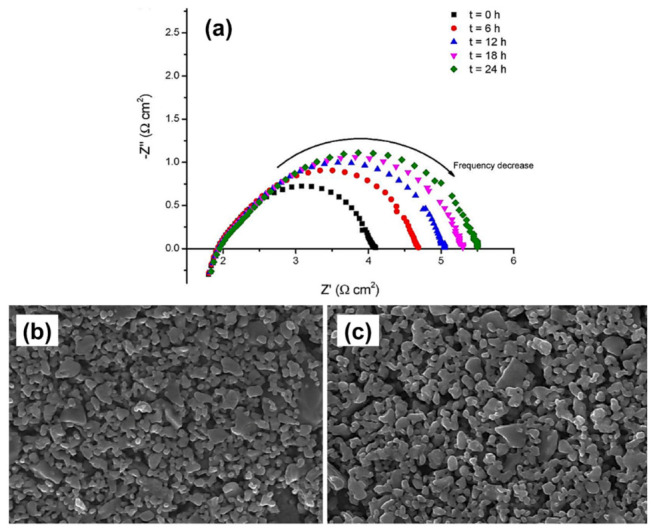
(**a**) EIS measurement of the LSCF cathode at 750 °C, XPS analysis, and SEM analysis (**b**) before and (**c**) after heat treatment time under open-circuit voltage [36] (^©^ The Electrochemical Society. Reproduced by permission of IOP Publishing. All rights reserved.).

Bucher et al. [39] examined the degradation behavior of the LSCF cathode as a function of water vapor and found the segregation and migration of Sr to a depth of greater than 150 nm and the formation of SrCrO_4_ and Cr_2_O_3_ species via the reaction with SrO on the cathode surface. Pan et al. [36] also studied the relationship between the performance degradation and compositional changes on the surface of the LSCF cathode by using electrochemical impedance spectroscopy (EIS) and XPS, respectively. The XPS analysis showed that the aged LSCF cathode exhibited significant segregation of Sr concentration and formation of SrO layer on the surface after the LSCF cathode was treated at elevated temperature (750 °C) for 24 h, thus remarkably decreasing the performance degradation as shown (Figure 7a) [36]. Figure 7a shows that the polarization resistance (*R*_P_) increases gradually, indicating the performance degradation of the LSCF cathode. Therefore, the degradation of the LSCF cathode is caused by the segregated Sr and the formation of the SrO layer on the surface, thus remarkably changing the composition at the surface.

**Figure 8:**
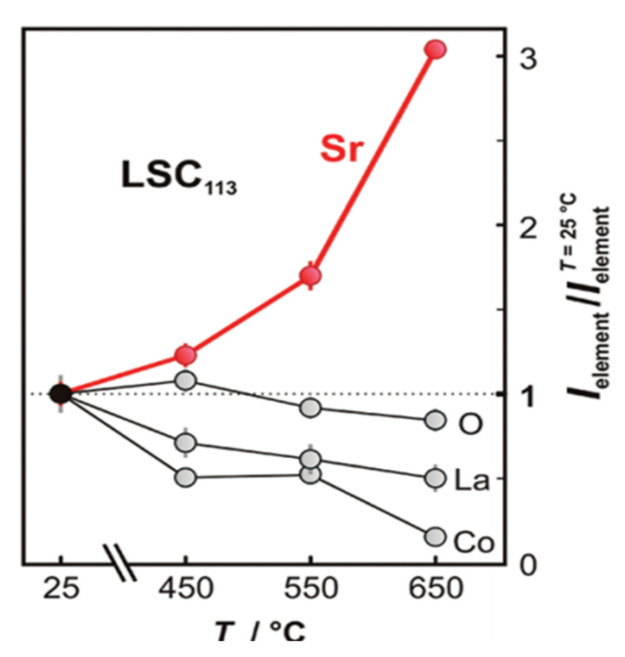
Increase in total Sr intensity with increasing temperature. (Reprinted with permission from [40], copyright American Chemical Society, 2012).

Vovk et al. [32] employed in situ XPS and reported a 5% increase in the Sr/(La + Co) ratio on the surface of the LSC thin-film after cathodic polarization. Mutoro et al. [40] also observed a threefold increase in the Sr 3d intensity and a decrease in the La 4d and Co 3p intensities upon heating the surface of the LSC thin film from 25 °C to 650 °C by using in situ synchrotron-based XPS. Therefore, Sr segregation or migration is favored during high-temperature treatment (Figure 8). Yu et al. examined the effect of Sr content on the Sr segregation and diffusion phenomena by using hard X-ray photoelectron spectroscopy and observed rich SrCO_3_ phase on the LSCF surface when the Sr content is increased [41].

The relationship between the extent of Sr surface segregation and the Sr content was successfully identified using surface-sensitive XPS analysis. Wang et al. [42] studied the LSCF symmetrical cell by using XPS (Thermo Scientific ESCALAB 205Xi, East Grinstead, UK) and observed a slight increase in the low-energy satellite peak of Sr 3d at 131.8 eV after heat treatment at 800 °C. Liu et al. [43] used XPS (Thermo VG Scientific Multilab 2000, East Grinstead, UK) and found that the Co concentration on the surface of the LSCF–Gd-doped ceria composite cathode increased from 10.66% to 19.65% (atomic percentage of cobalt) after cathodic polarization for 500 h at 750 °C. The XPS results reveal a correlation between the segregation and structural instability of LSCF cathodes.

XPS sputter depth profiling is also widely used to obtain elemental and composition information as a function of depth within layer and layer interfaces. DiGiuseppe et al. [31] used the depth profile capability of XPS to study the interfaces of the LSCF cathode up to a depth of approximately 50 µm after electrochemical testing [Figure 4iv] and detected Sr migration into the ceria barrier layer via surface diffusion. However, the Sr 3d peak does not change or shift with increasing depth. Therefore, no change occurred in the Sr chemical state and lack of formation of new compounds. Liu et al. [44] also investigated the migration of Sr and Co in LSCF after different cathodic polarization treatments at different depths by using XPS (Thermo VG Scientific Multilab 2000, East Grinstead, UK) and found that cathode polarization at 200 mA cm^−2^ promotes the migration of Sr and Co from the top surface to the bulk of the LSCF. This migration is responsible for suppressing the Sr surface segregation and performance activation of the LSCF cathode. The profile depth was set to 5–15 nm by eroding the LSCF surface through ion etching by using an argon beam with a diameter of 50 μm. Table 3 shows the Sr/La and Co/Fe ratio at different depths, and these values were calculated using the element ratios of the XPS pattern.

All Sr/La ratios after current polarization treatment (Sr/La = 0.67) are larger than the stoichiometric value of the LSCF, indicating the migration of Sr from the surface to the bulk of the LSCF cathode. After the current polarization treatment of 100 mA cm^−2^, the Sr/La ratio was four times higher than that of the LSCF before treatment, demonstrating the severe surface segregation of Sr. At high cathodic polarization (200 mA cm^−2^), the Sr/La ratios are lower than those of LSCF after 100 mA cm^−2^ treatment, demonstrating the diffusion of Sr from the surface into the bulk LSCF lattice. Hence, high cathodic polarization suppresses the formation of SrO on the surface of the LSCF cathode, resulting in improved ORR rate or low cathode polarization resistance. After cathodic polarization treatment, the Co/Fe ratios of the LSCF are lower than those of the untreated LSCF cathode, indicating the migration of Co from the surface to the bulk of the LSCF cathode. However, the migration of Sr and Co from the surface to the bulk of the LSCF cathode may weaken the mechanical stability by accommodating a substantial amount of oxygen vacancies in the LSCF structure [44].

Knöfel et al. [34] employed X-ray powder diffraction (XRD) and XPS to investigate the surface chemistry and phase stability of LSM/YSZ composite cathode under different fuel cell conditions. They observed that the degradation of LSM/YSZ composite cathode strongly depends on humidity, oxygen partial pressure, and heat treatment. The XRD results indicate the formation of secondary phases, such as strontium zirconate and lanthanum zirconate, at the LSM/YSZ interface caused by the surface diffusion and interaction of Sr and La with YSZ. This phenomenon is related to the increased concentration of Sr on the surface of the perovskite cathode, as confirmed by XPS analysis. Based on the ex-situ XPS analysis, the concentration of Sr cation on the surface increased, and this metal exists in the form of strontium carbonate or strontium hydroxide, which corresponds to high binding energy (133.3 eV). Monoclinic zirconia oxide (m-ZrO_2_) formed at the LSM/YSZ interface, indicating the activated inter-diffusion of Mn into YSZ at high temperatures.

**Figure 9:**
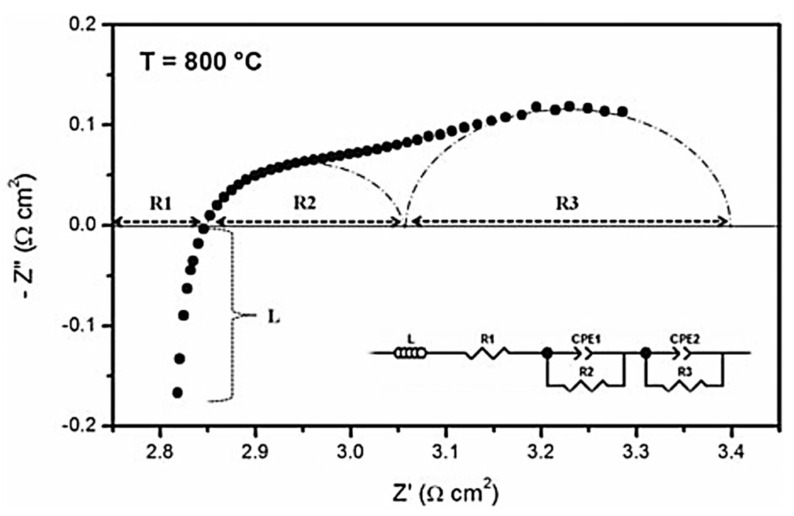
Impedance spectra of an LSCF symmetrical cell measured at 800 °C and equivalent circuit used to fit the impedance circuit (inset). (Reprinted with permission from Reference [48], copyright John Wiley and Sons, 2012).

These microstructural and compositional changes in the LSCF cathode could remarkably alter the electrochemical performance of the single cells. This phenomenon is possible because the electrochemical reactions for symmetrical cells with LSCF cathode consist of three physicochemical processes as follows: (1) electrolyte ohmic resistance, (2) charge transfer process associated with incorporation of O^2−^ ion at the cathode/electrolyte interface and electronic transfer at the interconnect/cathode interface, and (3) oxygen dissociation/adsorption on the cathode surface [45]. These processes occur at different times during charge transportation. The response time for electrolyte ohmic resistance is approximately zero [46]. EIS can be employed to analyze the above-mentioned rate-limiting steps in the cathode material. EIS can effectively characterize various electrochemical steps and simultaneously monitor electric and dielectric phenomena in the cathode material. The interfacial polarization resistance in the LSCF cathode was measured by applying constant-amplitude AC voltage over a predefined frequency on the symmetrical cell being investigated. The phase shift and amplitude of the resulting current response were measured as a function of frequency. If various rate-limiting steps occur at different frequencies, then the sum of all individual reaction steps represents the overall cathode reaction. The electrochemical reaction of cathode materials was measured using ASR, which is obtained via EIS analysis and was used to characterize the cathode symmetrical cell. A typical impedance spectrum of a cathode symmetrical cell at 800 °C is shown in Figure 9.

The interfacial polarization resistance in the LSCF cathode is associated with two processes, namely, oxygen molecule diffusion through the porous structure and charge transfer to reduce the oxygen molecules into oxide ions at the cathode/electrolyte interface [47]. The presence of two arcs in the impedance spectra indicates the two electrode processes (*R*_2_ and *R*_3_) during the ORR [48]. The impedance spectra were fitted with the equivalent circuit to investigate the rate-limiting steps in cathodes (Figure 9, inset). In the above-mentioned circuit, *L* is the inductance, *R*_1_ is the electrolyte ohmic resistance, *R*_2_ is the high-frequency arc that can be related to the charge transfer process associated with the incorporation of O^2−^ ions at the cathode/electrolyte interface, *R*_3_ is the low-frequency arc associated with oxygen dissociation/adsorption on the cathode surface, and CPE is the constant-phase element [49]. In general, low capacitance ranging from 10^−12^ F to 10^−6^ F is associated with electrolyte resistivity (bulk and grain boundary resistance), whereas high capacitance of >10^−5^ F is associated with electrode resistivity arc [50]. The CPE in the equivalent circuit can be expressed as follows [51]:

(2) $$Z_{\mathrm{CPE}}=1/{Q\;(j\omega )}^{n}$$

where *ω* = 2·*π*·*f·*(*f* is the frequency), *Q* is the pseudocapacitance, *j* is equal to $\sqrt{-1},$ and *n* is the empirical constant value between 0 and 1. The actual capacitance value can be calculated using Equation (3), as follows [24]:

(3) $$C=R_{p}^{\left(1-n\right)/n}\;Q^{(1/n)}$$

Mid- and low-frequency arcs for MIEC cathode materials can be attributed to the electrode process. LSCF cathodes can provide active sites for the ORR on the surface to the TPB and pathway for the incorporation/diffusion of O^2−^ ions through the bulk and at the cathode/electrolyte interface [52,53]. The overall ASR was calculated using the equation ${\mathrm{ASR}=(R}_{p}\times A)/2$, where *A* is the active area. The sum of the high- and low-frequency intercepts is the total interfacial or cathode polarization resistance (*R*_p_ = *R*_2_ + *R*_3_) of the symmetrical cell [54]. The interfacial polarization resistance of the LSCF cathode decreases gradually with increasing temperature.

## 4. Performance and Degradation Mechanism of LSCF Cathodes

### 4.1. Microstructural Degradation of the LSCF Cathode

**Figure 10:**
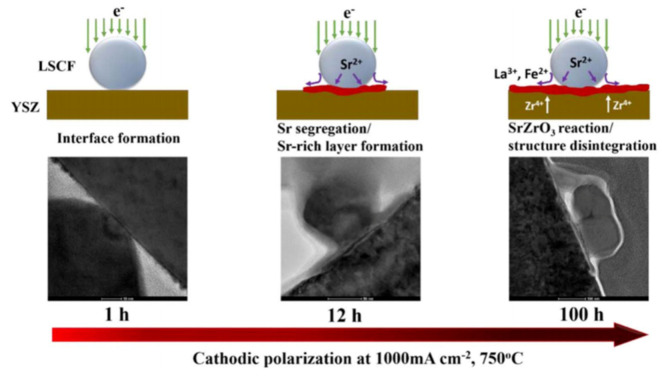
Scheme of the effect of polarization time on the Sr segregation and formation of SrZrO_3_ phase at the LSCF/YSZ interface region [56] (^©^ The Electrochemical Society. Reproduced by permission of IOP Publishing. All rights reserved).

The performance and long-term structural stability of the LSCF cathode under various SOFC operational conditions have been extensively studied [55]. Figure 10 shows the segregation of Sr cation and formation of insulating phase SrZrO_3_ at the LSCF/YSZ interface as a function of polarization time at 100 mA cm^−2^ and operating temperature of 750 °C [56]. This electrically insulating Sr-rich layer de-activates the oxygen diffusion kinetics and blocks the migration of oxygen ions at the cathode/electrolyte interface, thus affecting the electrical conductivities of the LSCF cathode [57,58]. It also alters the thermal expansion coefficient of the cathode/electrolyte layers, thereby affecting the mechanical stability of the interface [59]. This segregation or enrichment of cations is caused by the mismatch of the ionic radii between the dopant Sr^2+^ (1.44 Ǻ) and the host La^3+^ (1.36 Ǻ) [36,60]. Moreover, the electrostatic attraction between these ions produces a strong Columbic attraction that drives the dopant cations to the positively charged interface and causes the dopant Sr^2+^ cation to segregate toward the surface. At the LSCF surface, the concentration of Sr cations increases with the decrease in partial oxygen pressure and increasing operating temperature [61].

The segregation of cations, reactivity with YSZ, and deposition of SrO at the YSZ surface and the LSCF/YSZ interface has been studied as functions of oxygen partial pressure, air humidity, gas composition, temperature, and electrochemical polarization treatment [41,56]. Chen et al. [28,62] investigated Sr segregation and chemical reaction between deposited SrO and YSZ behavior under different cathodic polarization treatments at temperatures above 750 °C. The polarization treatment favored Sr segregation and diffusion to form an oxygen ion-blocking layer (SrZrO_3_ phase) in the LSCF/YSZ interface region. Under cathodic conditions, the segregation or enrichment of strontium (Equation (4)) and cobalt (Equation (5)) on the surface, the formation of SrZrO_3_ phase by reacting with yttria-stabilized zirconia (YSZ) electrolyte (Equation (6)), and the reactivity with chromium and contaminates (e.g., boron) (Equation (8)) can cause rapid deterioration of ORR kinetics and surface instability of LSCF-based cathode materials [37]. Under elevated operating temperatures, the segregation and/or structural distortion on the surface is enhanced, affecting the adsorption/desorption kinetics of the cathode.

(4) $${\mathrm{La}}_{0.6}{\mathrm{Sr}}_{0.4}{\mathrm{Co}}_{0.2}{\mathrm{Fe}}_{0.8}O_{3}\overset{\mathrm{cathodic}\;\mathrm{poloarization},\;750\;{}^{\circ}C}{\to }{\;\mathrm{La}}_{0.6}{\mathrm{Sr}}_{0.4-x}{\mathrm{Co}}_{0.2}{\mathrm{Fe}}_{0.8}O_{3}{+x\mathrm{SrO}}_{s}$$

(5) $${\mathrm{La}}_{0.6}{\mathrm{Sr}}_{0.4}{\mathrm{Co}}_{0.2}{\mathrm{Fe}}_{0.8}O_{3}\overset{\mathrm{cathodic}\;\mathrm{poloarization},\;750\;{}^{\circ}C}{\to }{\mathrm{La}}_{0.6}{\mathrm{Sr}}_{0.4}{\mathrm{Co}}_{0.2–y}{\mathrm{Fe}}_{0.8}O_{3}+y\mathrm{CoO}$$

(6) $${x\mathrm{SrO}}_{s}{+Y}_{0.08}{\mathrm{Zr}}_{0.92}O_{2}\overset{\Delta }{\to }{\mathrm{Sr}\mathrm{ZrO}}_{3}{+Y}_{2}O_{3}$$

(7) $${\mathrm{CrO}}_{3}+\mathrm{SrO}\to {\mathrm{SrCrO}}_{4}$$

(8) $${\mathrm{La}}_{0.6}{\mathrm{Sr}}_{0.4}{\mathrm{Co}}_{0.2}{\mathrm{Fe}}_{0.8}O_{3}{+\mathrm{CoO}+B}_{2}O_{3}\to {\mathrm{LaBO}}_{3}{+\mathrm{Fe}}_{2}O_{3}$$

### 4.2. Compositional Changes in LSCF Cathode

**Figure 11:**
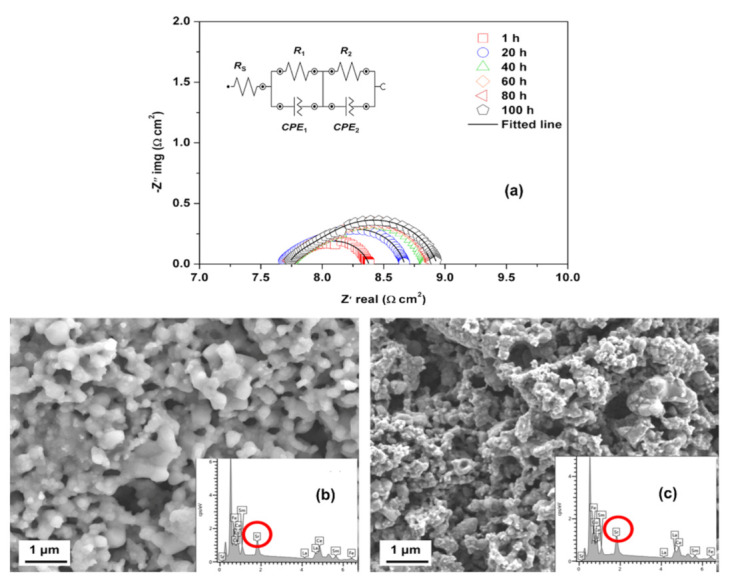
(**a**) EIS spectra as a function of thermal treatment time measured at 600 °C and FESEM surface images and EDX spectra of LSCF cathode (**b**) before and (**c**) after the thermal treatment. (Reprinted with permission from Reference [65], copyright Springer Nature, 2019).

The compositional change at the surface and bulk of the LSCF can be observed during the SOFC operating conditions. Hashimoto et al. [63] utilized atmospheric controlled high-temperature X-ray diffraction technique to study the thermo-chemical stability of cobalt-rich cathode material. They found that La_0.6_Sr_0.2_Co_0.8_Fe_0.2_O_3−δ_ decomposed completely into (La,Sr)_2_(Co,Fe)O_4_ and a CoO phase (halite) at relatively high oxygen partial pressure (ca. *P*_O2_ 10^−6^ bar at 800 °C). Wang et al. performed the elemental analysis of the LSCF cathode by using EDX and secondary-ion mass spectrometry; they found that the segregated Sr cation migrated to the YSZ electrolyte layer and reacted to form insulating SrZrO_3_ phase [64]. Ali et al. [65] employed field emission scanning electron microscopy (FESEM) coupled with EDX to investigate the microstructural stability and long-term performance degradation of the LSCF current-collecting layer cathode at 600 °C for over 100 h (Figure 11b,c); the Sr concentration on the surface increases from 7.4% to 10.3%. Moreover, the electrochemical polarization resistance increases gradually especially in the low-frequency region, where resistance is associated with the dissociation/adsorption of oxygen molecules and diffusion on the LSCF cathode surface (Figure 11a) [48]. The FESEM image shows substantial changes in the morphology and Sr segregation on the LSCF layer, which may be the main reason for the degradation of the performance of the LSCF cathode [65]. However, EDX provides a bulk Sr concentration and limited information about the speciation or oxidation state of each element in the samples.

The properties and reactivity of the surface depend on the bonding geometry of molecules to the surface, chemical composition, chemical structure, physical topography, atomic structure, and electronic state [66]. Various electron spectroscopic techniques have been employed to investigate all these properties and provide chemical information about the oxidation state and elemental composition of the surface of any solid substrate [67]. Surface-based spectroscopy is routinely used to obtain information on compositional changes near the surface region of the LSCF cathode; examples of this technique include XPS, ultraviolet photoelectron spectroscopy, Auger electron spectroscopy (AES), electron energy loss spectroscopy (EELS), reflected EELS, and high-resolution EELS. The long-term stability issues such as the Sr segregation and diffusion, formation of Sr-rich layer, and presence of secondary phases, such as SrZrO_3_ and SrCrO_4_ at the LSCF/YSZ interface region during the SOFC operation, which can accelerate the deterioration of the ORR and affect the electrochemical performance [41]. Thus, understanding the compositional changes associated with the segregation or enrichment of cations at the surface of the LSCF cathode is crucial. Thus, a characterization tool with high surface sensitivity must be utilized to understand the degradation mechanism in SOFC cathode materials.

## 5. Strategies to Suppress Sr Surface Segregation

As discussed in this review, XPS analysis is a promising technique to analyze the correlation between the segregation and performance degradation of the LSCF cathode material. Therefore, Sr segregation or enrichment of Sr on the surface of the LSCF significantly affects the compositional stability and subsequently causes detrimental effects on the surface property of the LSCF cathode because Sr is an extremely active compound in LSCF and can easily diffuse through the porous structure of the cathode material during high-temperature sintering and under cathodic polarization conditions. Therefore, Sr segregation is an inherent property of the LSCF cathode material, and it has been generally observed and unavoidable. Although the segregation of Sr cations cannot be prevented, it can be reduced via surface enhancement and the use of innovative nanostructured architecture through wet impregnation or infiltration. To increase the lifetime of an LSCF cathode, scholars must investigate its degradation mechanism under different operational conditions. Current research focuses on modifications on the cathode material, reaction barrier layer, and the interface between these two to improve the SOFC reliability and performance.

**Figure 12:**
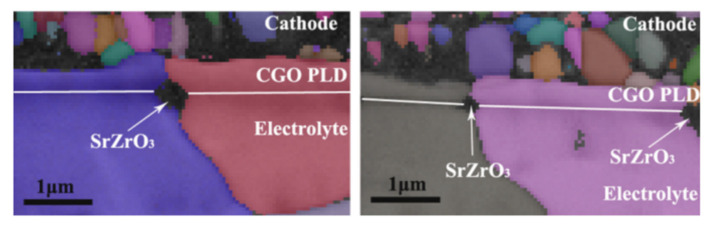
Electron backscattered diffraction image represents the typical migration behavior of segregated Sr from the surface of the cathode to form SrZrO_3_ phase between the CeO_2_ based barrier layer and the YSZ electrolyte. (Reproduced from Reference [70]. Copyright 2010 The American Ceramic Society).

Electrochemical polarization and fuel cell operating conditions significantly contribute to the formation of SrO and the migration of Sr cations to form an oxygen ion-blocking layer (SrZrO_3_ phase) at the cathode/electrolyte interface. Considerable efforts have been made to avoid surface segregation and interaction between the LSCF and YSZ to improve long-term structural stability and electrocatalytic activity for ORR. A dense barrier layer of the doped ceria-based electrolyte between LSCF and YSZ can be used to minimize the reaction between segregated Sr and YSZ under the cathodic polarization treatment (Figure 12). However, this approach cannot completely eliminate the formation of the resistive SrZrO_3_ phase at the LSCF layer and at the doped ceria/YSZ interface (Figure 12) because of the presence of an open porosity and the subsequent densification with the ceria-based interlayers at low sintering temperatures. In general, the GDC interlayer at the top of the pre-sintered YSZ electrolyte must be sintered below ~1200 °C to prevent GDC and YSZ from forming high-temperature chemical reactions. Densifying the interlayer at low sintering temperatures seems impossible because of the refractory aspect of ceria. However, this open porosity in the interlayers must be reduced to prevent the formation of an undesired chemical substance at the interlayer and the cathode/electrolyte interface and to restrict the Sr cation migration from the cathode to the zirconia-based electrolyte materials [68,69].

Numerous alternate deposition methods have been identified as important achievements for achieving a dense diffusion barrier interlayer, such as atomic layer deposition, PLD, MS, and chemical solution deposition. However, the cost of production to scale up the SOFC technology by using these approaches remains uncertain. The densification of the LSCF cathode is one of the important strategies to mitigate the migration of cations from the cathode to the interlayer/electrolyte interface layers. De Vero et al. [71] recently reported on the improved stability of the SOFCs by adopting a dense LSCF layer between the cathode–GDC interlayer interfaces. The thin and dense LSCF film prepared by the PLD between the porous LSCF cathode and the GDC interlayer effectively reduced the degradation process associated with the Sr cation migration. Fan et al. [72] stated that the LSCF cathode deteriorated less with an SDC interlayer between the electrolyte and the cathode using carbon-containing suspension plasma spray deposition along with lanthanum chromite coating. Uhlenbruck et al. [73] showed how CGO thin films formed a coat between the cathode and the electrolyte using physical vapor deposition (PVD), prevented the diffusion of strontium from the cathode, resulted in improved SOFC efficiency, and were more effective than screen-printed coatings. Roeder et al. [74] provided another method to improve degradation by modifying the LSCF cathode through atomic layer deposition with Group IVA oxides and cobalt-based compositions. These mitigating measures revealed that the segregated Sr migration is considerably suppressed from the LSCF to the GDC interlayer, thereby limiting the rapid formation of SrZrO_3_ after long-term activity. Therefore, the modification of electrode/interlayer/electrolyte layers can successfully reduce the interface resistance and consequently improve the cell efficiency and stability.

The stress/strain minimization of the surrounding lattice and electrostatic or charged interactions of the dopants is known as the main motivating factor for the isolation of Sr dopant cations to the surface of the LSCF cathode. This is attributed to the ionic radii imbalance between the dopant Sr^2+^ and host La^3+^ cations. However, this degradation mechanism in the LSCF cathode can be reduced or prevented via the surface modification through the doping of the high-valence element infiltration technique and adopting a nanostructured architecture using the wet impregnation or infiltration technique. Ding et al. [30] used the first principles density functional theory (DFT) computational method to locate and suppress Sr segregation in the LSCF cathode. Their study identified two thermodynamic driving forces (strain and surface charge) for Sr segregation and suggested to apply compressive strain and to reduce surface charge (reducing the concentration of surface oxygen vacancies) to reduce the Sr segregation. The compressive strain can be applied by the doping of larger elements and/or surface coating with catalytically active LSM phases in a porous LSCF substrate. The formation of excess oxygen vacancies on the surface can be reduced by doping high-valence elements in the Sr and B-site or low-valence elements in the La-site in order to suppress the Sr surface segregation.

Lynch et al. [75] modified the surface of LSCF cathode with a dense thin LSM surface coating by a solution infiltration process and tested at 700 °C under fuel cell operating conditions. The electrochemical performance showed that the cell with the LSM-infiltrated LSCF cathode exhibited improved stability toward Sr segregation with reduced interfacial polarization resistance. Giuliano et al. [76] also reported the beneficial effect of the LSM infiltration on the long-term stability and electrochemical performance of the LSCF cathode. This result suggests that the presence of the LSM thin layer on the LSCF backbone can inhibit the Sr segregation and increase the oxygen vacancy concentration when the cathodic overpotential is applied. This finding indicates the absence of an activation energy barrier for the oxygen dissociation/adsorption on the cathode surface, i.e., an increase in the oxygen exchange surface activity, and thus leads to the enhanced electrocatalytic activity for ORR [76]. Similar enhancement by the infiltration of a catalytic active phase on the electrocatalytic activity and the compositional stability of the LSCF cathode was also reported on La_0.8_Sr_0.2_FeO_3−δ_ [77], Sm_0.5_Sr_0.5_CoO_3−δ_ [78], La_2_NiO_4+δ_ [79], Pr_1−x_Sr_x_MnO_3_ [80], La_1−x_Sr_x_CoO_3−δ_, and La_1.97_Sr_0.03_Zr_2_O_7_ [81]. The infiltration or wet impregnation of catalytic active components into the LSCF backbone can also be carried out using a wet powder spray coating [82] and an inkjet printing technique [83].

**Figure 13:**
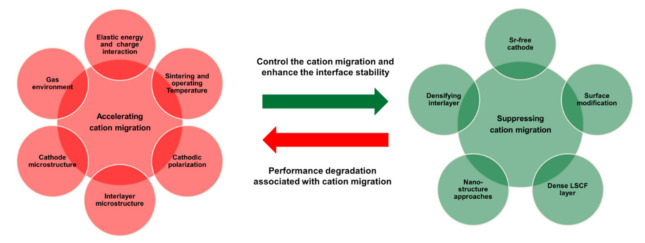
Factors influencing the cation migration and strategies to enhance the interface stability in the LSCF-based cathode materials for solid oxide fuel cells.

Doping with high-valence cations such as Nb^+5^, Sb^+5^, Mo^+6^, Ta^+5^, and Y^+3^ in the B-site is an effective strategy to stabilize the phase structure and the compositional changes of Sr-containing perovskite cathode materials. These large-sized cations tend to suppress the Sr segregation on the LSCF cathode surface by reducing the surface oxygen vacancies and influencing the ionic conductivity of the perovskite material [84]. Chen et al. [85] studied the effects of Nb cations at the B-site on the stability and electrochemical performance of LSCF-based cathode materials and observed remarkable improvement on the structural stability, leading to the minimized surface segregation of Sr cations. This high-valence addition of Nb^5+^ cations could reduce the valence state of the B-site cations and thus expand the LSCF lattice, which in turn can accommodate the large Sr^2+^ cations and thereby reduce the Sr segregation. This finding indicates that the introduced Nb dopant enhances the valence stability of the Co and Fe cations at the B-site, thus increasing the energy barrier and preventing the formation of excess oxygen vacancies at the surface. However, Nb doping exhibited a detrimental effect on the electrochemical activity of the LSCF cathode [86]. Dual doping of Nb and Pd into the B-site of the LSCF cathode can enhance the structural stability and the electrochemical performance because of the presence of highly active Pd nanoparticles in the LSCF structure that promote the ORR kinetics [86]. Figure 13 summarizes the factors influencing the cation migration in LSCF-based cathode materials and strategies to develop promising LSCF-based cathode materials for SOFCs and SOECs.

## 6. Concluding Remarks

The XPS, a powerful surface-sensitive technique for surface analysis, can be used to identify the elemental composition and chemical or oxidation state of samples near the surface region (1–10 nm). It has a wide range of applications, such as corrosion and failure analyses, and is a standard tool for surface material characterization from cookware coating to thin-film electronic and polymer surfaces. However, XPS has some limitations. It is very expensive, cannot detect hydrogen and helium, and has a slow processing time. For solid oxide fuel cells, XPS cannot profile the material in-depth as it is a surface technique. The active area of a porous LSCF cathode is best described as a buried interface with an extend of a few µm. XPS is a surface technique with a few nanometers of analysis depth and is therefore unsuitable for characterizing such systems. Furthermore, when characterizing LSCF cathodes using XPS, LSCF cathodes do not show electrochemical activity everywhere, but only in a localized zone (called the utilization length λ) close to the cathode/electrolyte interface. Typically, the value of the utilization depth is estimated to be around 5–20 μm for LSCF, depending on the operation conditions. As a result, researchers should proceed with extreme caution when reading the experimental details of research claiming to establish correlations between the chemical state at the (electrochemically inactive) surface of a porous LSCF cathode and the polarization resistance. Moreover, XPS requires a very high vacuum during analysis, meaning in situ characterization of SOFC is impossible using this technique.

Recent developments in XPS enable the measurement of the uniformity of elemental composition as a function of depth by ion beam etching or depth profiling. Angle-resolved XPS (ARXPS) also opens up new possibilities for measuring elemental composition as a function of depth by changing the sample tilt angle with reference to the analyzer. Unlike depth profiling XPS, ARXPS can carry out such measurements and in-depth analysis without ion beam sputtering. Furthermore, ARXPS is a non-destructive technique that measures the uniformity of elemental composition near-surface regions. The XPS is now available in ultra-high resolution for imaging and elemental analyses with spatial resolutions of as low as 70–100 nm. Spatially resolved XPS can analyze the full material at the nanoscale by adding high spatial resolution and imaging capabilities to XPS.

Near-ambient pressure XPS (NAP-XPS) is a relatively recent breakthrough that simplifies the investigation of SOFC materials. NAP-XPS is a less common method of XPS analysis that allows for the analysis of samples under realistic conditions at relatively high pressures (i.e., >2500 Pa). As a result, NAP-XPS does not require ultra-high vacuum (UHV) conditions in the analysis region because the sample is surrounded by a gas environment during the experiment. As a result, a wide range of samples, including insulating samples, biological samples, gases, liquids, and their interfaces, can be studied easily. When performing XPS measurements in a gaseous environment, the photoelectrons emitted by the samples are dispersed by collisions with the surrounding gas molecules before entering the hemispherical electron analyzer.

Given the development and implementation of photoelectron spectromicroscopy, the surface chemistry of the sample can be analyzed by full-field or scanning methods. The XPS has been widely used to study the degradation mechanism of SOFC cathode materials, which often affect the electrical performance and mechanical stability under cathodic polarization.

The LSCF cathode is one of the most popular and extensively studied materials for SOFC applications. However, scholars must investigate the degradation mechanisms of this material under different operational conditions to create an understanding of how to increase the lifetime of the LSCF cathode. Electrochemical polarization and fuel cell operating conditions significantly contribute to the formation of SrO and the migration of Sr cation to form an oxygen ion-blocking layer (SrZrO_3_ phase) at the cathode/electrolyte interface. Considerable efforts have been made to avoid surface segregation and interaction between the LSCF and YSZ to improve structural stability and electrocatalytic activity for ORR. A dense barrier layer of doped ceria-based electrolyte between LSCF and YSZ can be used to minimize the reaction between segregated Sr and YSZ under cathodic polarization treatment. However, this approach will not completely eliminate the formation of the resistive SrZrO_3_ phase at the LSCF layer and the doped ceria/YSZ interface. Applying compressive strain by surface coating with LSM material and reducing surface charge by doping with higher-valence elements in the Sr and B-site can minimize or prevent Sr surface segregation and enhance the structural stability and electrochemical polarization performance. Information about the surface property or surface chemistry of perovskite-type cathode materials is important because of differences between the chemistry of the surface and the bulk. Since XPS is a surface-sensitive characterization technique and only reveals the surface properties of the sample, it can be used to examine Sr and Co segregation on the surface of the LSCF cathode as a function of temperature, oxygen partial pressure, and electrochemical polarization to understand the potential degradation mechanism associated with the LSCF cathode. Thus, XPS technology enables new possibilities and opportunities for researchers to elucidate the degradation behavior of the perovskite cathode region (1–10 nm) near the surface.

## Figures and Tables

**Table 1:** Binding energy of electrons for selected elements in their elemental form.

Elements	Binding Energy (eV)								
	1s	2s	2p_1/2_	2p_3/2_	3s	3p_1/2_	3p_3/2_	3d_3/2_	3d_5/2_
La	38,925	6266	5891	5483	1362	1209	1128	853	836
Sr	16,105	2216	2007	1904	358.7	280.3	270	136	134.2
Ba	37,441	5989	5627	5247	1293	1137	1063	795.7	780.5
Sm	46,834	7737	7312	6716	1723	1541	1420	1110.9	1083.4
Gd	50,239	8376	7930	7243	1881	1688	1544	1221.9	1189.6
Y	17,038	2373	2156	2080	392	310.6	298.8	157.7	155.8
Zr	17,998	2532	2307	2223	430.3	343.5	329.8	181.1	178.8
Nb	18,986	2698	2465	2371	466.6	376.1	360.6	205	202.3
Tb	51,996	8708	8252	7514	1968	1768	1611	1276.9	1241.1
Pd	24,350	3604	3330	3173	671.6	559.9	532.3	340.5	335.2
Co	7709	925.1	793.2	778.1	101	58.9	58.9	-	-
Fe	7112	844.6	719.9	706.8	91.3	52.7	52.7	-	-
Ni	8333	1008.6	870	852.7	110.8	68	67.2	-	-
Cr	5989	696	583.8	574.1	74.1	42.2	42.2	-	-
Ca	4038.5	438.4	349.7	346.2	44.3	25.4	25.4	-	-
Mn	6539	769.1	649.4	638.7	82.3	47.2	47.2	-	-
O	531	22	-	-	-	-	-	-	-

**Table 2:** Binding energy levels of LSCF cathode under different cathodic polarization currents as determined by XPS.

Element	Binding Energy (eV)			
	Non-Treated	Open Circuit	100 mA cm^−2^	200 mA cm^−2^
La 3d5/2	832.97	832.58	833.45	832.71
Sr 3d5/2	131.78	131.66	131.54	132.45
Co 2p3/2	779.57	779.56	778.99	779.06
Fe 2p3/2	709.21	709.1	709.4	709.37
O 1s	531.33	531.56	531.24	531.41

**Table 3:** Sr/La and Co/Fe ratio determined by XPS for LSCF electrodes after polarization treatment at 750 °C in air for 120 h at different depths (Adapted from Ref. [44]).

Depth (nm)	Sr/La			Co/Fe		
	Non-Treated	100 mA cm^−2^	200 mA cm^−2^	Non-Treated	100 mA cm^−2^	200 mA cm^−2^
0	2.048	8.678	1.563	0.588	0.57	0.278
5	0.463	2.214	1.51	0.498	0.65	0.401
10	0.597	4.03	1.661	0.523	0.678	0.356
15	0.578	3.626	1.424	0.488	0.493	0.392

## Data Availability

Not applicable.
